# Effect of Divergent Genetic Selection for Growth on Spawning Quality in Gilthead Seabream (*Sparus aurata*)

**DOI:** 10.3390/ani15243527

**Published:** 2025-12-07

**Authors:** Cathaysa Pérez-García, Álvaro Lorenzo-Felipe, Shajahan Ferosekhan, Hyun Suk Shin, Sergi León-Bernabeu, Marisol Izquierdo, Daniel Montero, Rafael Ginés, Juan Manuel Afonso-López, María Jesús Zamorano

**Affiliations:** 1 Institute of Sustainable Aquaculture and Marine Ecosystems (IU-ECOAQUA), Universidad de Las Palmas de Gran Canaria, Taliarte, 35214 Telde, Spain; cathaysa.perez108@alu.ulpgc.es (C.P.-G.); alvaro.lorenzofelipe@ulpgc.es (Á.L.-F.); feroseaqua@gmail.com (S.F.); hyunsuk.shin@ulpgc.es (H.S.S.); sleonber@gmail.com (S.L.-B.); marisol.izquierdo@ulpgc.es (M.I.); daniel.montero@ulpgc.es (D.M.); rafael.gines@ulpgc.es (R.G.); juanmanuel.afonso@ulpgc.es (J.M.A.-L.); 2 Norwegian Institute of Food, Fisheries and Aquaculture Research (Nofima), 6600 Sunndalsøra, Norway

**Keywords:** spawning quality, genetic improvement, estimated breeding value, genetic selection, aquaculture, gilthead seabream

## Abstract

Gilthead seabream (*Sparus aurata*) is a prominent aquaculture species in Europe; however, the repercussions of growth-oriented selective breeding on reproductive performance under industrial conditions have not been adequately characterised. The present study evaluated the influence of divergent Best Liner Unbiased Prediction (BLUP)-based selection for low growth (LG) (*n* = 49; mean weigh ± SD = 842 ± 189 g) and high growth (HG) (*n* = 50; mean weight ± SD = 1127 ± 407 g) on spawning quality throughout the commercial mass-spawning season. A number of significant differences were detected between the genetic lines. The LG broodstock produced substantially higher oocyte yields and numbers of fertilised eggs (26% and 25% increases, respectively), indicating greater quantitative reproductive output. In contrast, the HG line exhibited marginally higher fertilisation, egg viability, and hatching rates, indicative of enhanced early developmental efficiency. Despite these contrasting patterns, both lines exhibited similar numbers of viable eggs, larvae, and comparable larval survival. These findings demonstrate that selection for growth impacts reproductive traits through different pathways: The selection of HG results in an enhancement of developmental performance, while the selection of LG leads to an optimisation of egg production. Across the spawning period, oocyte yield was identified as the primary driver of overall spawn quality. The findings of this study offer pertinent insights into the optimisation of broodstock management and the enhancement of sustainability and efficiency in gilthead seabream aquaculture.

## Simple Summary

The present study investigates the impact of genetic selection for growth rate on spawning quality in gilthead seabream (*Sparus aurata*). The study involved the comparison of two groups: a high-growth (HG) line and a low-growth (LG) line. These groups were monitored throughout the spawning season under real production conditions. The results demonstrated that the LG line exhibited a higher reproductive capacity, with a greater number of eggs and fertilised oocytes. In contrast, the HG line demonstrated marginally superior biological efficiency, exhibiting modest enhancements in fertilisation and egg viability. Nevertheless, the final number of live larvae in both groups was comparable. These findings may assist in the optimisation of genetic selection strategies, with the aim of enhancing both growth and reproductive performance. This, in turn, could contribute to a more efficient and sustainable seabream production.

## 1. Introduction

Total aquaculture production of seabream (*Sparus aurata*) in Europe and the rest of the Mediterranean in 2023 is estimated at 332,966 t, according to statistics from APROMAR, FEAP, and FAO. It is among the top 10 species produced with a 12.1% year-on-year rate of change [1].

Gilthead seabream production is carried out through the integration of its biological cycle into a production chain in aquaculture companies. This chain comprises hatchery companies, which serve both as reservoirs of selected elite broodstock (nucleus) and producers of juveniles (multipliers). These juveniles are then transferred to sea cages or land-based estuaries (fattening companies) to be finally marketed essentially in ration size (traders) [2].

Although considerable progress has been made in the aquaculture industry, hatchery conditions are still far from ideal, resulting in frequent challenges that entail significant economic losses [3]. On an industrial scale, the efficiency of hatcheries depends on the correct integrated management of all production factors that affect the quality of their spawn, which in turn conditions correct body development, internally and externally [4,5]. Among these factors, survival and body development stand out for their effects on the other production strata [6,7,8]. Thus, to improve spawn quality, companies establish spawns from batches of breeders consisting of between 30 and 60 animals, at a density of between 4 and 5 kg/m^3^ [9]. The constitution of broodstock through phenotypic selection prevents knowledge of the animal’s genealogy and predisposes them to increased inbreeding in breeder batches [10], which also leads to lower selection accuracy [11]. So far, it is estimated that only 50% of companies use genetic criteria for the constitution of breeding stocks under well-established selection programmes [12].

Spawning quality, measured in terms of fecundity, viability, and hatching rates, has been the subject of numerous studies. It has been shown that, even with low male ratios, it is possible to maintain high egg quality over several breeding seasons under controlled conditions [13]. Complementarily research on Peruvian grunt, *Anisotremus scapularis*, has highlighted the importance of egg and larval quality in the consolidation of captive stocks [14].

To control the sexual maturity of broodstock and have spawn available throughout the year, companies organise their batches by inducing spawning using photo-thermal manipulation [15,16], thus allowing them to satisfy the demand of fattening companies. In this way, hatcheries always have batches of active breeders in the spatial–temporal window with the highest spawning quality (Cristóbal Aguilera, personal communication), which is affected by different genetic, nutritional, and management factors.

Among the management and nutritional factors that can affect spawning quality in seabream, studies have found improvements in the quality and viability of seabream eggs, especially when a sex ratio of 1:1 in terms of biomass is maintained [17]. Thus, two males per female are used as females mature at higher weights (three years of age), while males mature at two years. Seabream broodstock fed 1.6% n-3 HUFA (highly unsaturated fatty acids) had better spawn quality in terms of fecundity, hatching, and larval survival rates. In particular, the n-3 HUFA content of eggs showed a positive correlation with dietary n-3 HUFA content, mainly due to changes in the 20:5 n-3 eicosapentaenoic acid (EPA) content of eggs [18]. Furthermore, recent studies in gilthead sea bream pointed out that selenium supplementation showed improvements in reproductive performance (total number of eggs, fertilisation, hatchability) [19]. Other authors have described the regulatory effect of vitamin E on seabream spawning quality. Thus, an increase in dietary α-tocopherol levels of breeders reduced the percentage of abnormal eggs and increased fecundity [20]. A study on the relationship between spawning quality and the age of spawning females of gilthead seabream showed that the estimated values of relative fecundity, fecundity, hatching rates, and egg diameter were influenced by the timing of spawning (early, middle, or late) and the age of the females [21]. Thus, three-year-old females were found to be more fertile and to have a higher fecundity than older females, with a significantly different Arachidonic Acid (AA)/EPA ratio depending on the time of spawning.

As genetic factors, it has been evaluated how genetic selection for growth in gilthead seabream broodstock affects their reproductive performance, including spawning quality, under different feeding regimes during spawning plateau (three months) [22]. These authors found that broodstock selected for high growth (HG) and fed a fish oil (FO) diet had significantly better sperm, egg, and larval quality. These findings suggest that genetic selection for growth may have a positive impact on progeny quality in seabream. Also, the existence of genotype and environmental interactions (genetic selection and diet) for different spawning quality traits, such as total number of eggs, fertilised eggs, viable eggs, hatched larvae, and 3-day post-hatching (dph) larvae [23], has also been described.

On the other hand, the impact of the genetic background of deformities in the broodstock selected by their Estimated Breeding Value (EBV) for deformities or normal morphology on the quality of spawning throughout the spawning period is also examined [8]. It has been demonstrated that deformed broodstocks exhibit shorter spawns, reduced egg production, and a lower number of live larvae in comparison to normal broodstocks. Moreover, the number of live larvae emerged as the most significant spawn quality trait in breeders fed a regular commercial diet [8].

The objective of this study was to examine the impact of divergent selection on the quality of spawning in gilthead seabream, throughout the entire spawning season and under industrial rearing conditions. The estimation of EBV was performed for broodstock low and high growth to commercial size.

## 2. Materials and Methods

The present study was carried out with third-generation breeders of the Spanish National Breeding Program (PROGENSA^®^), selected for growth at harvest size [24]. The experiment was approved by the Bioethics Committee of the University of Las Palmas de Gran Canaria (REF: OEBA_ULPGC_24/2019), in accordance with the European Union directive on the protection of animals for scientific purposes (2010/63/EU).

### 2.1. Biological Material

A total of 5449 gilthead seabream adults from the Canary Islands region were evaluated for the weight at commercial size/age (EBVw) using a linear model with the software VCE 6.0 [25,26]. Genotyping was also established using the microsatellite supermultiplex (SMsa1) [27] with the GENEMAPPER (v.5.0) software (Applied Biosystem^®^, 5791 Van Allen Way, Carlsbad, CA 92008, USA). Parental assignment was carried out by comparing breeders and descendants’ genotypes using the software VITTASIGN v.8.2 [28].

Two batches of breeders were selected based on their EBVw, gender, and additive relationship coefficient (*a*), and according to the optimal contribution selection method [29]. The breeders’ EBVw selected had an amplitude of 63.71 g. The low growth broodstock (LG) consisted of 49 animals (22 females and 27 males) with average EBVw and standard deviation (SD) values of −28.67 ± 40.03 g, and the high-growth broodstock (HG) consisted of 50 animals (18 females and 32 males) with an average EBVw and SD of +35.04 ± 44.26 g. The biomass–sex ratio (kg male/kg female) was similar between batches, at 1.5 for LG and 1.4 for HG (Table 1), following other authors’ recommendations [3,17]. Breeders (males and females) were the same age, 3 years old. Male and female growth-related traits (weight and length) were larger in HG than in LG broodstock (Table 1).

The fish were distributed in two 10 m^3^ tanks (one tank for LG, and another one for HG) at the facilities of the Institute of Aquaculture and Sustainable Ecosystems of the University of Las Palmas de Gran Canaria (IU-ECOAQUA). All tanks were supplied with 16 L/min of seawater at a pH of 7.98, with 37 g L^−1^ salinity, 19.3 °C mean temperature, and 7.5 ppm mean oxygen concentration. The two batches’ breeders (LG and HG) were not replicated because, along the spawning quality period in gilthead seabream, there is no significant effect between replicates within treatments [16,30,31,32].

Thus, breeders’ maturation and spawning occurred spontaneously under natural conditions at the latitude of the Canary Islands [15,33]. There were no breeder losses during the spawning season.

Fish were fed at 1% of body weight, twice daily (9:00 and 14:00 h) using a diet (crude protein: 58.9%, crude lipid: 21.3%, and ash: 9.8%) produced by Skretting ARC (Burgos, Spain) with all requirements for the spawning period.

### 2.2. Evaluation of Spawning Quality

**Figure 1:**
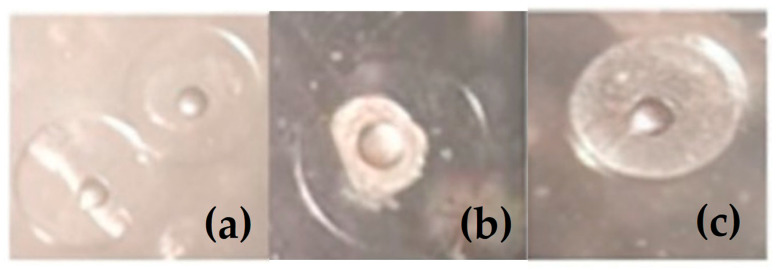
Illustration of observed egg types: (**a**) viable eggs, (**b**) non-viable egg, and (**c**) an un-fertilised egg.

To assess the quality of the spawn, floating eggs were collected daily during the spawning period using a 500 μm net placed at the side outlet of the tank. The eggs were subsequently transferred to 10 litre containers of tank water with strong aeration. Once the sample was well homogenised, 10 mL was pipetted out three times. Two 10 mL samples and five 5 mL samples were taken for counting in a Bogorov chamber and observation under a binocular stereo microscope. The 5 mL samples were counted twice, and then the average of the three replicates was calculated. Spawning quality parameters were calculated, such as the total number of fertilised, viable, and hatched eggs and larvae produced per kg female [18,34,35], as described in the AQUAEXCEL-ATOL approach, including oocyte yield (ATOL:0001723), fertilisation rate (ATOL:0001775), viability rate (ATOL:0001531), hatching rate (ATOL:0001531), and larvae survival rate (ATOL:0001531), which were estimated volumetrically after counting the samples daily. Furthermore, utilising two 96-well plates containing 192 floating eggs (one per well) with 200 μL of filtered seawater and incubated at 19 °C, the number of alive larvae was counted to calculate the hatching rate (number of hatched larvae/numbers of fertilised eggs), and larvae survival rate (%). Figure 1 illustrates the morphology of viable eggs (a), non-viable eggs (b), and un-fertilised eggs (c). The hatching and survival variables were measured following the assessment of two 96-well ELISA plates for hatching after 24 h and one day post hatching (1 dph), and for survival, at 48 h and three days post hatching (3 dph). [36,37]. The number of fertilised, viable, and hatched eggs, as well as the number of live larvae, were calculated as associated values, as the industry is to collect quantitative and qualitative data.

### 2.3. Statistical Analysis

In order to compare the evolution of spawn quality in both LG and HG selection broodstock, all traits were analysed during the entire spawning season. The evaluation period was from 1 February to 7 June 2019. To facilitate data comparison, all spawning quality traits were grouped by fortnight based on the estimated daily values and the effect of the time period on the spawning season (see Table 2).

All data were analysed for normality and homoscedasticity using the Shapiro–Wilk and Levene tests, respectively. When the data did not follow a normal distribution, a logarithmic transformation was performed on the direct qualitative variables and an arcsine transformation on indirect qualitative variables. If non-normality persisted, the data were analysed using non-parametric Kruskal–Wallis tests [38].

Thus, a comparison was made between the two genetic lines (LG vs. HG) within fortnights. Furthermore, an analysis of the effect between fortnights (from F4 to F12) within the genetic background was conducted, following Scheffé’s post hoc analysis, after applying an analysis of variance (ANOVA) (viable eggs and larvae survival rate for LG) to the general linear model, which is specified as follows:

Y*_ij_* = µ + α*_i_* + ε*_ij_* (1)

where µ is the population mean, α*_i_* is the fixed factor *i* (genetic background or fortnight), and ε*_ij_* is the residual error associated with the value *ij*.

To explain the trait number of live larvae 3 dph within the fortnights, a statistical algorithm was utilised to perform a standard regression analysis and estimate the variance components of principal factors. The predictor traits under consideration were oocyte yield, fertilisation rate, viability rate, hatching rate, and larvae survival rate.

Statistical analysis was performed using the IBM SPSS Statistics for Windows, version 22.0 (IBM Corp). Significant differences were considered at the 0.05 confidence level.

## 3. Results

The spawning period of the experiment was from December 2018 to June 2019 (Table 2). The initial three fortnights of spawning were excluded from the analysis of the results, as they correspond to the acclimatisation of the broodstock once the batches had been created. Thus, the evaluation of the total spawning period was over a 108-day period, grouped into nine fortnights. The spawning traits of both genetically selected lines were evaluated over the course of an entire reproductive season.

As presented in Table 3, the mean values of the directly estimated spawn quality traits (oocyte yield, fertilised eggs, viable eggs, hatched eggs, and number of alive larvae) are reported, along with the indirect estimates (fertilisation rate, viability rate, hatching rate, and larval survival rate) (As illustrated in the Supplementary Materials). These values are reported per genetic line and within fortnights per genetic line.

### 3.1. Comparison Between the Total Production of the Two Divergent Selection Genetic Lines

The traits of oocyte yield and fertilised eggs exhibited significant differences between the two divergent selection lines, being higher in the LG line than the HG line (25.81 and 25.13% higher, respectively). Regarding fertilisation, viability, and hatching rates, there were statistically significant differences between the genetic lines, but in this case, the HG line exhibited higher values compared to the LG line, with increases of 1.5%, 3.9%, and 2.9%, respectively. However, no significant differences were observed between the two genetic lines with regard to viable eggs, hatched eggs, number of live larvae, and larval survival rate.

### 3.2. Comparison of Spawning Quality Traits Between Fortnights Within and Between Divergent Selected Genetic Lines

In order to better understand and define spawning quality, all traits examined were analysed using a fortnight as the unit for grouping daily spawns. This approach enabled the fortnight to function as a factor of variation, both between genetic lines within each fortnight (rows) and across fortnights within each genetic line (columns) (Table 3).

In the fortnightly comparison of the LG line, oocyte yield values were maximum at the beginning of the spawning season and decreased to a minimum at the end of assessment. The Kruskal–Wallis non-parametric test showed that there were no significant differences between the first fortnight (from 4 to 9), with an average production of 35,834 eggs per kg female, gradually decreasing to around 7000 eggs per kg female in the last fortnight.

The analysis by fortnight of the HG line showed a similar dynamic to the one described in the LG line, with the first seven fortnights producing on average a similar number of eggs, with no statistically significant differences observed (26,246 eggs/kg female). However, in the subsequent two fortnights, the lowest egg production was recorded (7770 eggs/kg female).

The fortnightly analysis of the oocyte yield showed significant differences only in the fourth and sixth fortnights. The LG line had a higher total number of eggs in all cases (Table 3).

The values of fertilised eggs (Table 3) within both LG and HG lines follow a very similar pattern to the oocyte yield in the different fortnights. In the case of the LG line, no significant differences were recorded for the first seven fortnights, with an average production of fertilised eggs of around 32,611 fertilised eggs/kg female in these fortnights. At the end of the spawning season, this number decreased to its lowest level in the last fortnight, with only 7000 eggs per kg female. In the HG line, the average production over the first seven fortnights was 23,000 eggs per kg female. Production gradually decreased from the ninth fortnight onwards, with around 6000 fertilised eggs produced in the final evaluated fortnight (Table 3).

In the case of viable eggs, when analysing the values per fortnight for the LG line, the results revealed that the maximum occurred in F6 with a total of 27,640 viable eggs/kg female (Table 3). This trait exhibited a decline in the last two fortnights. The first six analysed fortnights showed no statistically significant differences in the LG line. The HG line follows a similar pattern to that described for LG, with, in this case, the maximum in F7 and a total of 25,490 eggs viable eggs/kg female (Table 3). No difference was found when comparing both selection lines for this trait (Table 3).

The highest values for hatched eggs and the number of live larvae were recorded during the middle fortnight, specifically in F6 and F8, respectively, for the LG line and in the F8 for the HG line. The maximum values obtained for each line did not demonstrate statistical differences, although the HG line exhibited higher values (32,290 hatched eggs in HG vs. 26,430 hatched eggs in LG or 27,340 live larvae in HG vs. 18,060 live larvae in LG) (Table 3). Within the same genetic line, a consistent pattern was observed in both traits: low values at the beginning, high values during the middle fortnights, and low values at the end (Table 3).

Fertilisation, viability, hatching, and larval survival rates did not show statistical differences within the LG line over the fortnights analysed (Table 3). Notably, the highest values recorded in fortnights F7 and F9 were found to be statistically different from those observed in F4, F5, and F11, which exhibited the lowest hatching rates. The fortnights that showed marked differences for larval survival rates in HG were F4 and F7.

### 3.3. Relationships Between Spawning Quality Traits

**Figure 2:**
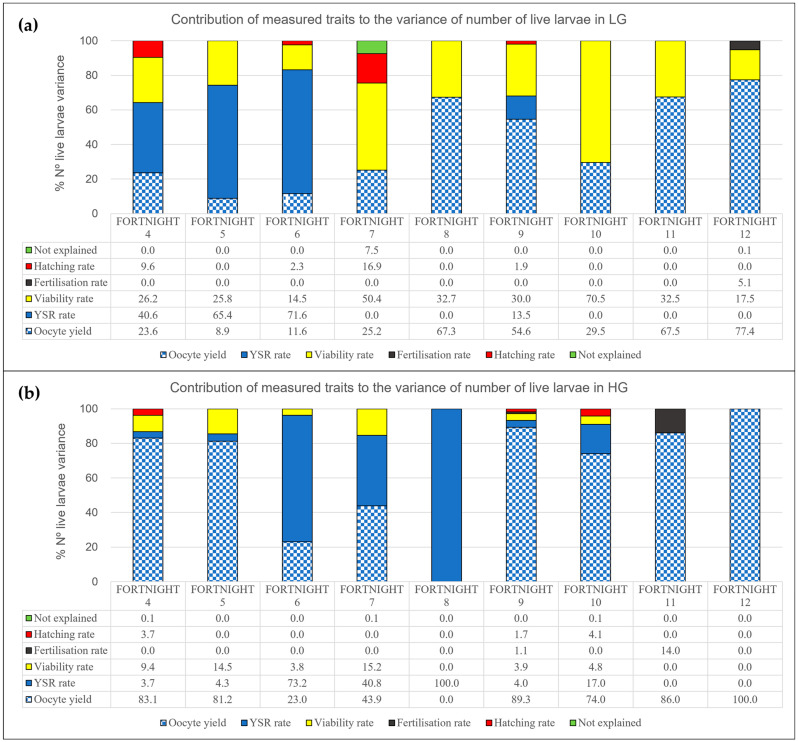
Graphical representation of the percentage explained by each of the evaluated traits (oocyte yield, fertilisation rate, larvae survival rate, hatching rate, viability rate) in the variance of the number of live larvae. (**a**) Histograms of the variance for LG line. (**b**) Histograms of the variance for HG line.

Figure 2a,b shows the importance of the oocyte yield, fertilisation rate, larvae survival rate, hatching rate, and viability rate on the number of live larvae.

The trait oocyte yield explains the majority of variance in the total number of live larvae. The influence of oocyte yield occurs in 45% of the fortnights when looking at the results of the LG line, and even more so in 78% of the fortnights in the HG line. It was found that the maximum influence was reached in the last fortnight for both the LG and HG line.

As illustrated in Figure 2a, other traits, such as larval survival, accounted for 65 and 72% in fortnights 5 and 6, respectively, in the LG line. As illustrated in Figure 2b, the HG line provides a comprehensive explanation of the total variance observed in fortnight 8.

## 4. Discussion

In the present study, the impact of divergent genetic selection for growth on the spawning quality of gilthead seabream, under industrial aquaculture conditions, is reported for the first time. Spawning quality traits (oocyte yield, fertilised eggs, viable eggs, hatched eggs, number of live larvae, fertilisation, viability, hatching and larval survival rates), over the entire natural spawning period (from December to June, including the acclimatisation period), are described in detail. It is widely acknowledged that the quality of gametes, in terms of both quantity and quality, is paramount for the competitiveness and efficiency of aquaculture enterprises [8,39,40,41,42].

The oocyte yield and fertilised eggs traits show the highest variability, with differences between the two genetic lines (LG and HG). Comparing these results for both traits with those of other authors, we can see that there are few studies in which the entire spawning period has been evaluated. The evaluation of spawn quality during the entire reproductive period in batches of breeders with a deformity background, based on their genetic evaluation by EBV [8], reported considerably higher values of oocyte yield and fertilised eggs than those described in this study, for animals that were normal. However, the values for both traits in deformed individuals are lower than those described by us in both selected lines. In contrast, the LG line demonstrated the highest values in the fortnightly analysis, thereby corroborating the findings of other researchers on gilthead seabream with a distinct genetic background and with n-3 LC-PUFA levels in their diet [22]. This finding is consistent with the observations reported in groups of subjects who were fed diets with nutritional disparities, particularly those consuming diets analogous to industrial diets (which include fish oil). The total number of egg production for each of lines (LG and HG) in this study is consistent with the published data on gilthead seabream, as reported by previous researchers [14,19,21]. The values are higher in the first two spawning periods of the animals (3 years old), to which both stocks of this study belong. Furthermore, this allows us to state that the differences in production are not due to the effect of age [21], as both batches are three years old.

Other parameters related to biological efficiency such as fertilisation, viability, and hatching rates demonstrated significant differences between the lines. The HG line exhibited elevated levels of these parameters. As asserted by other authors [22,23], the practice of genetic selection for high growth has been demonstrated to yield eggs that exhibit superior fertilisation, viability, and hatchability rates. This finding serves to reinforce the prevailing notion that reproductive performance is determined not solely by the quantity of eggs, but also by the quality of these ova. Despite the higher egg production of the LG line, the HG line is superior and consequently more profitable for the industry, thereby ensuring optimal flock growth and viability. Conversely, it is acknowledged that the efficacy of this hatching rate does not appear to be attributable to any effect of temperature, which is consistent with the observations reported by other researchers on gilthead seabream [33,43].

The larvae survival rate from both selected lines was found to be equivalent. Recent studies conducted on other fish species that also have genetic selection programmes, such as rainbow trout (*Oncorhynchus mykiss*), have revealed a phenotypic correlation between larval survival in the early stages and female fertility [44]. In addition, the genetic architecture of early survival traits was investigated under family genetic models in salmonids species [45,46], concluding that the heritability of early survival was low-to-moderate and that maternal environmental effects were important. This maternal influence has been documented in research on wild fish of Atlantic cod (*Gadus morhua*) [47]. In gilthead seabream, to the best of our knowledge, these estimates are not available, among other reasons, because of the dynamics of industrial-level production, which is carried out through mass spawning. However, the parameters previously referenced were utilised to undertake a comparative analysis of the spawn of animals exhibiting disparate genetic origins with respect to deformity or normality [8]. The findings indicated that the values ranging from 88 to 90% for hatching exhibited no significant disparities, a finding that aligns with the results obtained for the high-growth line in the present study. However, a marginal elevation in these values was observed for the low-growth line, with the viability ratio approximating 60%. It has been documented that the larvae survival rate did not show significant differences according to the selection for growth in the parents [23]. Nevertheless, other studies [8] have reported divergent findings. In particular, it was observed that normal animals exhibited a higher survival value than those found in crosses of animals with a genetic background associated with deformity. In the present study, larval survival was found to be higher in animals selected for high growth. This suggests that the selection for growth applied in the majority of breeding programmes does not appear to affect the quality of the clutch according to this particular parameter. Although all parameters should be evaluated as a whole, the number of total eggs did show significant differences in favour of the low-growth line, potentially indicating a superior adaptation of the LG line to reproductive characteristics or traits as opposed to those associated with fattening. However, a significantly higher percentage of dead eggs was observed in the LG line compared to the HG line, underscoring the necessity to quantify the various variables to ascertain the quality of the eggs [40] and spawning [41].

A number of studies have demonstrated a positive correlation between deformities, weight and length at different stages, larval or juvenile [48,49,50,51]. The range of values for European seabass, *Dicentrarchus labrax*, is from 0 to 0.40 [48] and from 0.07 to 0.13 [49]. For Atlantic cod, *Gadus morhua*, the value is 0.50 [50], and for gilthead seabream, it is from 0.20 to 0.46 [51]. This may provide a rationale for the observed congruence between the outcomes of the oocyte yield and the live larvae in the context of our study, in comparison to the findings of the research undertaken on a select group of gilthead seabream [8].

The evolution of spawning over the fortnightly period and the evolution of the yield of oocytes and fertilised eggs are of particular interest. The highest values appear at the beginning and decrease during the spawning period. A similar pattern in both traits for fish with normal and deformed backgrounds has previously been described in other works on the same species [8].

According to the present study, the number of live larvae is predominantly influenced by oocyte yield, across both the LG and HG genetic lines. However, within the HG line, this factor proved to be more salient throughout all fortnights. The viability rate represented the third most relevant spawn quality trait in both the LG and HG lines. The results obtained in this study are consistent with the broader patterns documented in the existing literature [18] concerning the mass spawning of broodstock in the same geographical location, and with respect to fortnights [8].

Future studies analysing the family contribution in breeding stock selected for high growth compared to those destined for low growth could provide valuable information on the influence of genetic improvement programmes on the family composition of selected animals. In this regard, it is important to maintain an optimal balance between the number of contributing families and the minimization of inbreeding, with the aim of preserving maximum genetic talent while maintaining the effectiveness of selection programmes.

## 5. Conclusions

Divergent selection for growth in gilthead seabream influences spawning quality under industrial conditions, with high-growth lines enhancing early developmental efficiency and low-growth lines maximising egg production. Oocyte yield is the key determinant of overall spawn quality, highlighting its importance for the optimisation of broodstock management and the enhancement of aquaculture efficiency.

## Figures and Tables

**Table 1:** Description of the broodstock of the gilthead seabream (*Sparus aurata*) selected for the mass spawning experiment in the low-growth (LG) and high-growth (HG) genetic lines (mean ± SD).

Broodstock Features	LG	HG
Male weight (g)	891.35 ± 246.42	1039.84 ± 288.73
Male length (cm)	33.54 ± 2.98	34.94 ± 2.78
Female weight (g)	793.04 ± 131.89	1213.95 ± 524.5
Female length (cm)	32.34 ± 1.56	35.85 ± 4.17
Male biomass (kg)	33	24
Female biomass (kg)	22	17
Total biomass	55	41
Biomass rate, male: female	1.5	1.4

**Table 2:** Fortnights established throughout the spawning season from 2018 December to 2019 June, including the specific days involved in acclimatation and evaluation period.

	Fortnights	Code	Period
Acclimatation			
	1, 2, 3	F1, F2, F3	From 29th December 2018 to 31st January 2019
Evaluation			
	4	F4	From 1st to 15th February 2019
	5	F5	From 16th to 28th February 2019
	6	F6	From 1st to 15th March 2019
	7	F7	From 16th to 31st March 2019
	8	F8	From 1st to15th April 2019
	9	F9	From 16th to 30th April 2019
	10	F10	From 1st to15th May 2019
	11	F11	From 16th to 31st May 2019
	12	F12	From 1st to 7th June 2019

**Table 3:** The mean values of spawning quality traits throughout the spawning season by fortnight (oocyte yield, fertilised eggs, viable eggs, hatched eggs, live larvae number, fertilisation rate, viability rate, hatching rate, and larval survival rate) per genetic line (LG, low growth; HG, high growth).

Trait	Genetic	F4	F5	F6	F7	F8	F9	F10	F11	F12	Total Spawning Season
Oocyte yield (×10^3^)	LG	38.24 ± 3.03 ^abA^	38.82 ± 4.11 ^ab^	36.74 ± 1.64 ^aA^	31.62 ± 2.72 ^abc^	33.75 ± 2.70 ^ab^	27.45 ± 2.32 ^abcd^	23.15 ± 1.86 ^bcd^	15.53 ± 3.00 ^cd^	7.07 ± 1.76 ^d^	29.44 ± 1.27 ^A^
	HG	25.78 ± 3.38 ^abB^	28.82 ± 4.34 ^a^	27.89 ± 2.60 ^aB^	31.33 ± 2.50 ^a^	28.01 ± 2.31 ^a^	23.11 ± 2.97 ^abc^	18.78 ± 1.86 ^abc^	9.12 ± 1.96 ^c^	6.42 ± 1.06 ^bc^	23.40 ± 1.19 ^B^
Fertilised eggs (×10^3^)	LG	38.01 ± 3.01 ^aA^	38.52 ± 4.08 ^aA^	36.08 ± 1.86 ^aA^	31.41 ± 2.72 ^ab^	33.50 ± 2.72 ^a^	27.78 ± 2.32 ^abc^	22.98 ± 1.84 ^abc^	15.36 ± 2.96 ^bc^	7.01 ± 1.75 ^c^	29.17 ± 1.26 ^A^
	HG	25.71 ± 3.38 ^abB^	28.73 ± 4.33 ^aB^	27.73 ± 2.57 ^aB^	31.21 ± 2.50 ^a^	27.89 ± 2.31 ^a^	23.00 ± 2.97 ^abc^	18.70 ± 1.85 ^abc^	9.08 ± 1.95 ^c^	6.37 ± 1.05 ^bc^	23.31 ± 1.19 ^B^
Viable eggs (×10^3^)	LG	25.06 ± 3.17 ^ab^	22.60 ± 2.53 ^abc^	27.64 ± 1.44 ^a^	17.99 ± 2.97 ^abc^	22.32 ± 2.26 ^abc^	15.33 ± 1.99 ^abcd^	12.27 ± 2.24 ^bcd^	10.03 ± 2.57 ^cd^	2.49 ± 0.91 ^d^	18.34 ± 1.04
	HG	21.58 ± 3.16 ^ab^	24.64 ± 4.56 ^a^	23.86 ± 1.98 ^a^	25.49 ± 2.47 ^a^	23.18 ± 2.09 ^a^	19.09 ± 3.24 ^abc^	15.41 ± 1.60 ^abc^	6.38 ± 1.54 ^c^	4.39 ± 0.38 ^bc^	19.33 ± 1.11
Hatched eggs (×10^3^)	LG	22.93 ± 3.27 ^ab^	20.53 ± 1.97 ^abc^	26.43 ± 1.13 ^a^	17.21 ± 3.14 ^abc^	20.86 ± 2.12 ^abc^	13.47 ± 2.09 ^bc^	10.88 ± 2.16 ^bc^	9.81 ± 2.89 ^bc^	2.92 ± 1.04 ^c^	17.61 ± 1.01
	HG	19.72 ± 2.87 ^ab^	22.71 ± 4.39 ^a^	22.32 ± 2.37 ^a^	24.49 ± 2.56 ^a^	32.29 ± 6.98 ^a^	25.52 ± 7.28 ^ab^	14.58 ± 1.54 ^ab^	5.97 ± 1.61 ^b^	4.64 ± 0.15 ^ab^	20.93 ± 1.67
N° live larvae (×10^3^)	LG	17.61 ± 2.83 ^a^	12.32 ± 2.84 ^ab^	17.80 ± 1.57 ^a^	12.53 ± 2.02 ^ab^	18.06 ± 1.89 ^a^	10.19 ± 1.47 ^ab^	9.17 ± 1.90 ^ab^	7.92 ± 2.70 ^ab^	1.66 ± 0.40 ^b^	13.04 ± 0.85
	HG	18.07 ± 2.88 ^a^	20.23 ± 1.72 ^a^	15.46 ± 2.86 ^ab^	16.26 ± 2.34 ^ab^	27.34 ± 6.83 ^a^	19.85 ± 5.50 ^ab^	10.88 ± 1.52 ^ab^	4.65 ± 1.46 ^b^	3.34 ± 0.69 ^ab^	16.78 ± 1.52
Fertilisation rate (%)	LG	99.38 ± 0.14	99.24 ± 0.25 ^B^	97.74 ± 1.23 ^B^	99.12 ± 0.27 ^B^	99.17 ± 0.20	99.32 ± 0.10	99.31 ± 0.20	98.99 ± 0.35 ^B^	99.17 ± 0.40	98.11 ± 0.93 ^B^
	HG	99.68 ± 0.06	99.67 ± 0.16 ^A^	99.46 ± 0.22 ^A^	99.64 ± 0.06 ^A^	99.55 ± 0.10	99.43 ± 0.09	99.58 ± 0.08	99.71 ± 0.16 ^A^	99.30 ± 0.40	99.57 ± 0.05 ^A^
Viability rate (%)	LG	65.64 ± 7.14 ^B^	61.59 ± 6.17 ^B^	75.61 ± 12.70 ^B^	54.34 ± 6.41 ^B^	65.78 ± 3.35 ^B^	56.47 ± 5.77 ^B^	50.95 ± 8.15 ^B^	56.45 ± 6.99	47.81 ± 10.95	60.31 ± 2.15 ^B^
	HG	83.93 ± 3.74 ^A^	81.08 ± 7.05 ^A^	86.97 ± 2.84 ^A^	80.46 ± 5.36 ^A^	82.69 ± 2.44 ^A^	79.06 ± 6.59 ^A^	82.63 ± 2.62 ^A^	72.26 ± 7.82	71.73 ± 5.60	80.79 ± 1.71 ^A^
Hatching rate (%)	LG	90.37 ± 4.12	92.14 ± 2.46	96.23 ± 1.36	91.38 ± 4.06 ^B^	93.79 ± 1.85	89.02 ± 3.33 ^B^	87.03 ± 3.13	84.51 ± 4.44	86.59 ± 1.51 ^B^	90.72 ± 1.08 ^B^
	HG	92.06 ± 1.50 ^b^	88.54 ± 3.73 ^b^	91.48 ± 5.50 ^ab^	98.44 ± 0.30 ^aA^	95.48 ± 1.87 ^ab^	98.83 ± 0.40 ^aA^	94.87 ± 1.73 ^ab^	92.42 ± 1.89 ^b^	95.66 ± 2.56 ^abA^	94.22 ± 0.91 ^A^
Larval survival rate (%)	LG	78.04 ± 5.94	56.14 ± 9.69 ^B^	67.21 ± 5.30	74.76 ± 2.76	86.26 ± 2.08	78.20 ± 3.33	83.86 ± 3.68 ^A^	70.23 ± 8.42	67.86 ± 10.89	74.25 ± 2.05
	HG	90.33 ± 2.98 ^a^	87.80 ± 4.48 ^abA^	66.71 ± 8.14 ^ab^	66.96 ± 5.31 ^b^	80.40 ± 3.77 ^a^	75.54 ± 4.00 ^ab^	71.23 ± 4.39 ^abB^	71.62 ± 5.35 ^ab^	70.55 ± 16.77 ^ab^	76.37 ± 1.87

Results are expressed as means ± SEM. For each treatment, values in the same column followed by different capital letters are significantly different (*p* < 0.05) between genetic lines during the same spawning period. Values in the same row followed by different lowercase letters are significantly different (*p* < 0.05) within each genetic line between fortnights. In the final column, the values in the same column followed by different capital letters are significantly different (*p* < 0.05) between genetic lines in the total spawning quality. For each treatment, values in the same column followed by different capital letters are significantly different (*p* < 0.05) between genetic lines, LG, Low Growth; HG, High Growth.

## Data Availability

The datasets generated and analysed during the current study (Excel files and SPSS outputs) are available from the corresponding author upon reasonable request.

## Supplementary Materials

The following supporting information can be downloaded at: https://www.mdpi.com/article/10.3390/ani15243527/s1, Figure S1: Evolution of spawning quality and quantity in genetic lines, low growth (LG) and high growth (HG) by fortnight during the spawning period; Figure S2: Evolution of spawning quality and quantity in genetic lines, low growth (LG) and high growth (HG) by fortnight during the spawning period.

## Abbreviations

The following abbreviations are used in this manuscript:

AA	Arachidonic Acid
BLUP	Best linear unbiased prediction
DPH	Days post hatching
EBV	Estimated Breeding Value
EPA	Eicosapentaenoic acid
FO	Fish oil
HG	High growth
LG	Low growth
n-3 HUFA	Highly unsaturated fatty acids
