# Supplementary material for: Effect of Divergent Genetic Selection for Growth on Spawning Quality in Gilthead Seabream (Sparus aurata)
Source: Animals (Basel). 2025 Dec 7;15(24):3527. doi: 10.3390/ani15243527 (PMC12729285; doi:10.3390/ani15243527)
Supplement: Supplementary file 1 [file animals-15-03527-s001.zip › animals-3995862-supplementary.pdf]

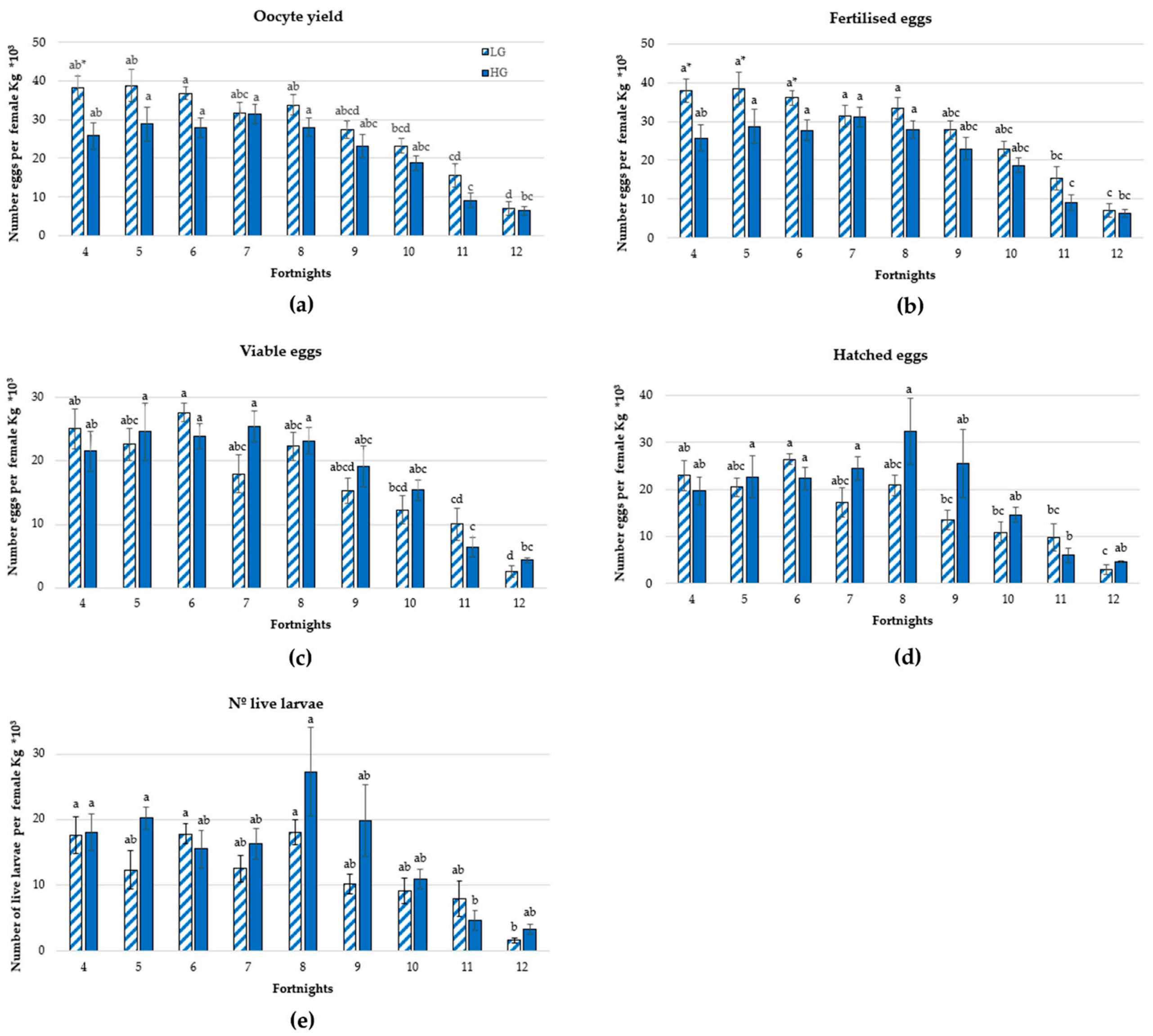

Figure S1. Evolution of spawning quality and quantity in genetic lines, low growth (LG) and high growth (HG) by fortnight during the spawning period. (a) oocyte yield, (b) fertilized eggs, (c) viable eggs, (d) hatched eggs, (e) number of live larvae. Means bearing different superscript letters differ significantly ($p < 0.05$). Values in the same column followed by asterisk are significantly different ($p < 0.05$) between genetic lines (LG, HG).

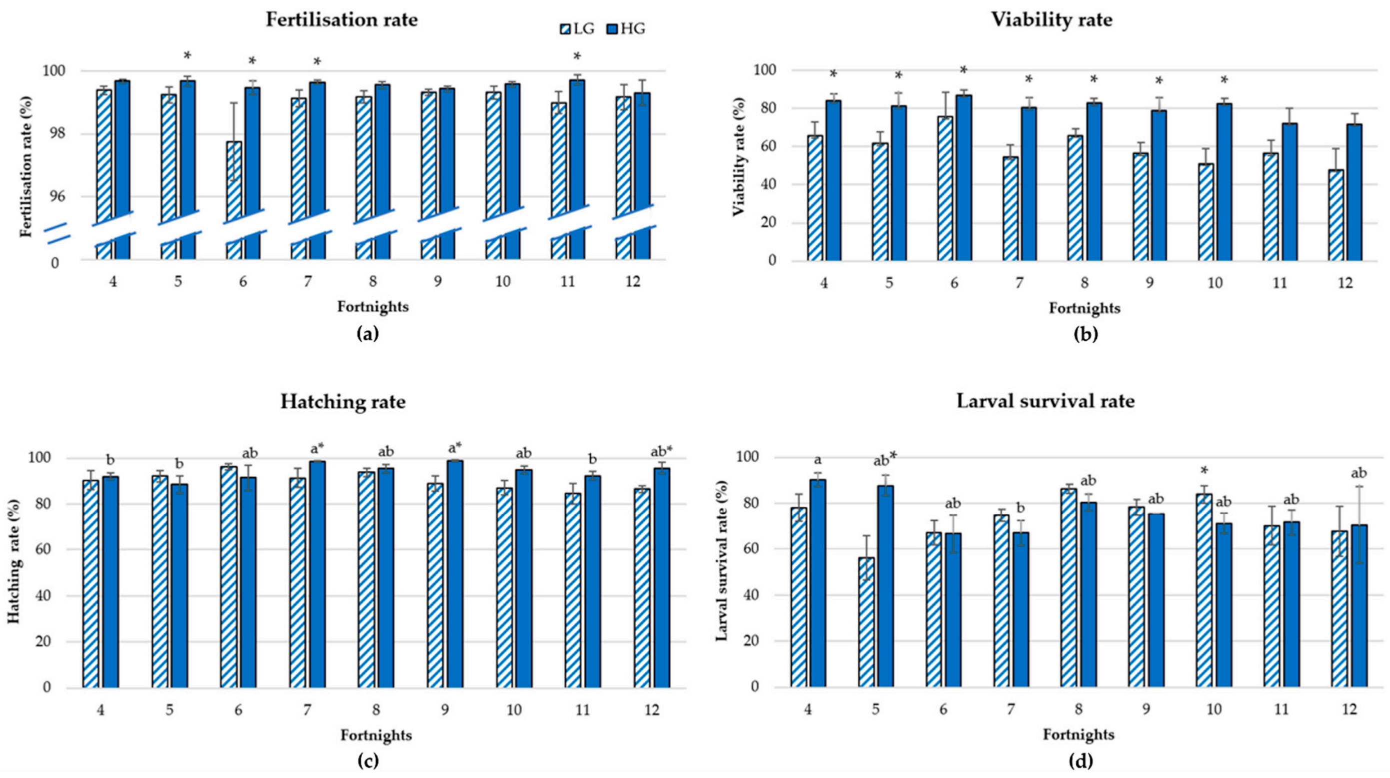

Figure S2. Evolution of spawning quality and quantity in genetic lines, low growth (LG) and high growth (HG) by fortnight during the spawning period. (a) fertilization rate, (b) viability rate, (c) hatching rate, (d) larval survival rate. Means bearing different superscript letters differ significantly (p < 0.05). Values in the same column followed by asterisk are significantly different (p < 0.05) between genetic lines (LG, HG)
